# The Impact of Mental Health Stigma and Ageism on Students’ Intention to Work with Older Adults: A Mixed Methods Design

**DOI:** 10.1093/geroni/igab046.2317

**Published:** 2021-12-17

**Authors:** Cindy Woolverton, Katelind Biccum, Aiping Yu, Lanlan Xin, Jessica Strong

**Affiliations:** 1 Michael E. DeBakey VA Medical Center, Houston, Texas, United States; 2 University of Prince Edward Island, University of Prince Edward Island, Prince Edward Island, Canada; 3 University of Prince Edward Island, Charlottetown, Prince Edward Island, Canada

## Abstract

Approximately 20% of older adults have a mental or neurological disorder which can cause significant disability. With a growing older adult population, there is a need for providers receiving specialized training in aging to provide quality care. However, there continues to be shortages of students seeking careers in geriatrics and especially in working with older individuals with mental health (MH) concerns. The present study explored the relationship between MH stigma, ageism and intention to work with older adults among undergraduate students. Undergraduate students (N=188) completed a battery of questionnaires including intention to work with older adults, positive and negative attitude towards older adults, and open-ended questions exploring MH stigma views. Regression results indicated that MH stigma, positive, and negative attitudes significantly predicted intention to work with older adults, (F(3, 182) = 8.51, p = .000). Examination of the coefficients revealed that positive attitudes significantly predicted intention to work with older adults (t=4.38, p=.000), and MH stigma demonstrated a trend towards significance (t=1.90, p=.059). Open-ended responses were analyzed using qualitative description methods which revealed themes consistent with negative and positive stereotypes, MH problems going undetected, and need for additional support in recognizing and treating MH conditions among older adults. Positive attitudes are an important predictor in students’ intention to work with older adults, and MH stigma may be an important factor to explore further. Qualitative themes also describe how MH concerns are an important area to focus on among older adults, although there continues to be evidence of aging stereotypes.

